# Co-Pyrolysis for Pine Sawdust with Potassium Chloride: Insight into Interactions and Assisting Biochar Graphitization

**DOI:** 10.3390/ma16103667

**Published:** 2023-05-11

**Authors:** Linen Xie, Liangcai Wang, Jianbin Zhou, Huanhuan Ma

**Affiliations:** 1 Joint International Research Laboratory of Biomass Energy and Materials, Co-Innovation Center of Efficient Processing and Utilization of Forest Resources, College of Materials Science and Engineering, Nanjing Forestry University, Nanjing 210037, China; 2 College of Materials Science and Engineering, Nanjing Forestry University, Nanjing 210037, China

**Keywords:** pine sawdust, KCl, co-pyrolysis, activation energy, graphitization

## Abstract

This effort aimed to explore the activation and catalytic graphitization mechanisms of non-toxic salts in converting biomass to biochar from the perspective of pyrolysis kinetics using renewable biomass as feedstock. Consequently, thermogravimetric analysis (TGA) was used to monitor the thermal behaviors of the pine sawdust (PS) and PS/KCl blends. The model-free integration methods and master plots were used to obtain the activation energy (E) values and reaction models, respectively. Further, the pre-exponential factor (A), enthalpy (ΔH), Gibbs free energy (ΔG), entropy (ΔS), and graphitization were evaluated. When the KCl content was above 50%, the presence of KCl decreased the resistance to biochar deposition. In addition, the differences in the dominant reaction mechanisms of the samples were not significant at low (α ≤ 0.5) and high (α ≥ 0.5) conversion rates. Interestingly, the lnA value showed a linearly positive correlation with the E values. The PS and PS/KCl blends possessed positive ΔG and ΔH values, and KCl was able to assist biochar graphitization. Encouragingly, the co-pyrolysis of the PS/KCl blends allows us to target-tune the yield of the three-phase product during biomass pyrolysis.

## 1. Introduction

Biomass is abundant, clean, and renewable, so great efforts have been made to develop and utilize biomass energy to achieve the “dual-carbon” goal [1,2,3]. Pyrolysis can convert biomass feedstock into biochar through an advanced conversion mode [4]. As is known, the specific surface area, cation exchange capacity, electrical conductivity, and pH values of biochars synthesized by pyrolysis affect their application [5,6]. Of these, the specific surface area is one of the most critical parameters affecting their performance. Unfortunately, biochars prepared by pyrolysis have low specific surface areas, which limits their use in many applications (e.g., electrode materials, catalysts, and adsorbents). As an example, Cen and co-workers produced rice straw-based biochars by pyrolysis, and the specific surface area of these rice straw-based biochars was in the range from ~68 m^2^/g to ~99 m^2^/g [7]. Similarly, Wang et al. [8] obtained coconut shell-based biochar with a specific surface area of 160 m^2^/g by pyrolysis of coconut shells. The specific surface areas recorded for the mentioned biochars were below 200 m^2^/g, which makes them under-valorized.

To solve the above problems, activation has aroused growing attention because the existence of activators can enhance the specific surface area [9,10]. Accordingly, a series of activators for activation processes have been investigated, including H_3_PO_4_ [11], KOH [12], and ZnCl_2_ [13]. However, KOH and ZnCl_2_ can pollute the environment, which limits the large-scale production of activated carbon. To match the activation process with the green development, non-toxic salts (e.g., NaCl and KCl) have been gradually used as activators in recent years with the following virtues: (1) non-toxic, low-corrosive, easily removed by water, recoverable from waste liquids, and suitable for large-scale preparation; and (2) have a template effect and can produce pores with regular pore size [14,15]. Unfortunately, the mechanism of interaction between non-toxic salts and biomass, especially how non-toxic salts affect the activation energy (E), pre-exponential factor (A), enthalpy (ΔH), Gibbs free energy (ΔG), and entropy (ΔS) of biomass during the activation process, has not been fully revealed. As is known, co-pyrolysis can assist in understanding the interaction of two matters. For instance, Li et al. [16] investigated the catalytic behavior of alkali metals and alkaline earth metals during the pyrolysis of lignocellulosic biomass. The results indicated that the dehydration capacity of alkaline earth metals induced the repolymerization between phenolic products. Similarly, Saynik et al. [17] studied the effect of alkali and alkaline earth metals on in situ catalytic fast pyrolysis of lignocellulosic biomass. The results suggested that alkaline earth metals accelerated pathways and increased yields of thermally derived char and non-condensable gases. Encouragingly, the activation process can be considered as the co-pyrolysis of biomass/activator blends under a nitrogen atmosphere. This is a highly critical fact as it allows us to evaluate the mechanism of activator–biomass interaction from the perspective of pyrolysis kinetics.

By comparison, in addition to the specific surface area, electrical conductivity is one of the most valuable parameters of biochar, especially when it is used as an electrode material [18,19]. As is known, the electrical conductivity of biochar is mainly influenced by its graphitization. Good electronic conductivity provides a low resistance channel for electron transport [20]. Further, extremely high temperatures are usually employed to enhance the graphitization of biochars [21,22]. Unfortunately, extremely high temperatures also result in the collapse of the pore structure of biochars and a significant decrease in yield; in other words, the specific surface area and graphitization are mutually constrained. Catalytic graphitization is an effective strategy to improve the conductivity of carbon materials [23]. Among these, metal (Fe, Co, and Ni) salts are particularly effective as catalysts for producing graphitized structures in carbon materials at temperatures below 1000 °C. This catalytic process makes it possible to convert non-graphitized precursors into graphitized carbon, thus expanding its applicability to a broader range of applications [24,25]. However, to the best of our knowledge, few studies have systematically focused on the mechanism of catalytic graphitization. It is well known that the mechanisms of interaction between two biomasses at elevated temperatures are usually unraveled using the co-pyrolysis method [26]. Interestingly, the catalytic graphitization of biochar represents a biomass/catalyst interaction under elevated temperatures. A variation consists of the replacement of one of the biomasses with a catalyst. This leads us to believe that the method used to study the co-pyrolysis mechanism of the two biomasses can be used to investigate the action mechanism of catalytic graphitization. Thus, this work aimed to explore the activation and catalytic graphitization mechanisms of non-toxic salts in converting biomass to biochar from the perspective of pyrolysis kinetics using renewable biomass as feedstock.

As such, low-cost and highly widely available pine sawdust (PS) and non-toxic KCl were used as raw materials for the case study. To reveal the interaction mechanism between PS and KCl, the weightless behaviors of PS, KCl, and PS/KCl mixtures were evaluated by thermogravimetric analysis (TGA). Moreover, the reaction model recorded for the samples was evaluated using Criado master plots [27]. Finally, the A, ΔH, ΔG, and ΔS values were studied using the obtained E values. Of note is that the lnA varies linearly with apparent activation energy. The appropriate amount of KCl improved the graphitization of the pyrolysis residue by reducing the E, ΔS, and ΔH values of the PS/KCl blends.

## 2. Experimental Section

### 2.1. Material Preparation

Pine sawdust (PS) samples were sourced from Jiangxi province in China, washed and dried, then ground into powder samples with a particle size ranging from 45 to 71 μm. Potassium chloride (analytical grade), namely, KCl, was supplied by Sinopharm Chemical Reagent (Shanghai, China). Mixing of KCl with PS was undertaken using the solid phase ball-milling method. Herein, the PSKCl-21 sample was obtained via mixing PS and KCl featuring a 2:1 weight ratio. The PSKCl-11 sample was manufactured by mixing PS and KCl in a 1:1 weight ratio. Similarly, the PSKCl-12 sample was fabricated by mixing PS and KCl in a 1:2 weight ratio. Alternatively, the principal symbols and acronyms are exhibited in Abbreviations.

### 2.2. Thermogravimetric Analysis

Weightless behavior of samples was analyzed using thermogravimetric analysis (TGA) in an N_2_ atmosphere. More information regarding the thermogravimetric (TG) analyzer is available in a previous work [28]. The temperature was gradually raised from room temperature to 850 °C by steps of 10, 20, 30, 40, and 50 °C/min, sequentially. Finally, the weightless behaviors of the samples were determined. Additionally, the calculation formulas of the reaction kinetics, activation energy, reaction model, and thermodynamic parameters are recorded in a previous work [29]. In addition, Raman spectroscopy (DXR532) with a wavelength of 532 nm was employed to analyze the defect or graphitization information of samples.

## 3. Results and Discussion

### 3.1. Weightless Behavior

**Figure 1:**
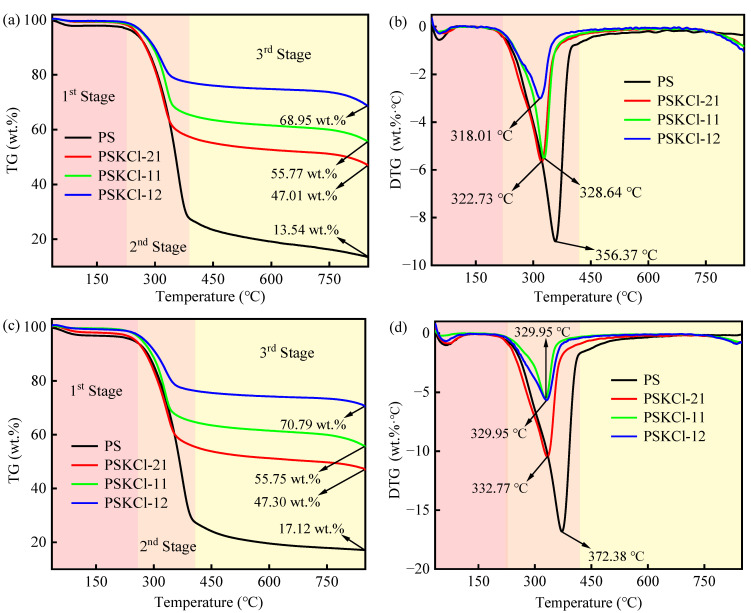

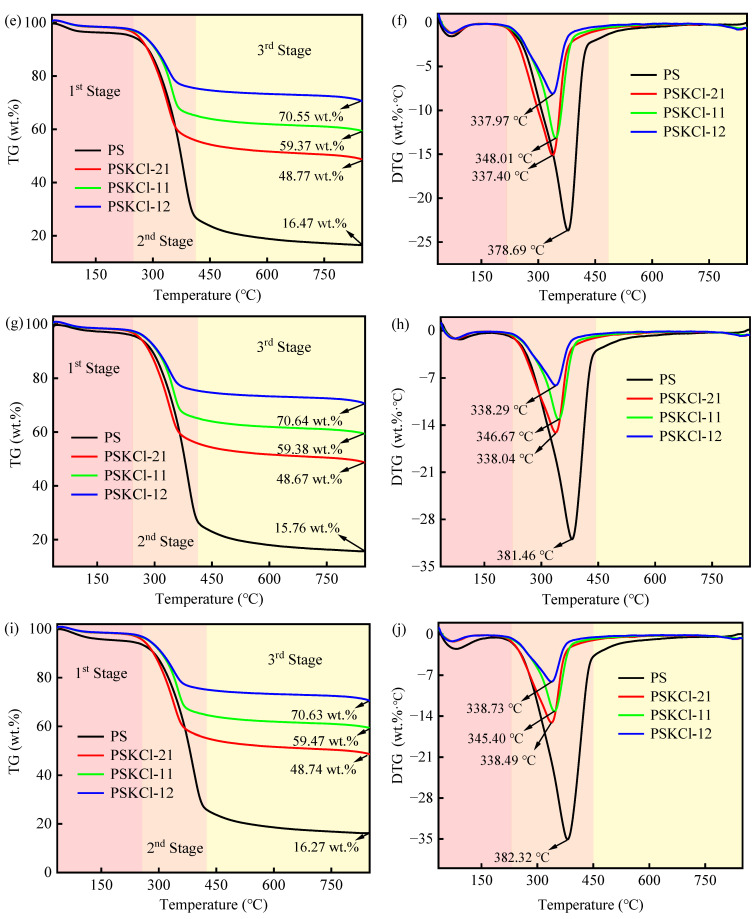
TG curves of 10 °C/min (**a**), 20 °C/min (**c**), 30 °C/min (**e**), 40 °C/min (**g**), and 50 °C/min (**i**); DTG curves of 10 °C/min (**b**), 20 °C/min (**d**), 30 °C/min (**f**), 40 °C/min (**h**), and 50 °C/min (**j**).

Figure 1a–j reveals the weightless behaviors of the samples at 10, 20, 30, 40, and 50 °C/min. Noticeable differences were found in the weightlessness behaviors of the samples. As an example, at 10 °C, the second stage of PSKCl-12 ended significantly earlier than that of PS. In addition, the pyrolysis residue weight (13.54 wt.%) of PS was considerably lower than that of PSKCl-12 (68.95 wt.%). Regarding PS, the pyrolysis process was composed of the dry dehydration stage (45–226 °C), pyrolysis stage (226–390 °C), and carbonization stage (>390 °C). Therein, dry dehydration represents a slow-weight-loss stage, and the second stage was the fast generation stage of small molecule compounds [30]. Consequently, the sample exhibited an outstanding weight-loss peak in the second stage (Figure 1b). That is, 180–450 °C could be defined as the pyrolysis temperature range of the sample, and this result was similar to that recorded for rice straw and rice husk [31,32]. Notably, all curves (Figure 1b,d,f,h,j) presented a weight-loss peak in the first and second stages, regardless of the heating rate and composition of the samples, which was similar to other biomasses (e.g., corn straw, wheat straw, and grape stem) that presented a weight-loss peak in the first and second stages [28,33,34]. As expected, the size of the weight-loss peaks and the time of appearance varied, suggesting that the component content influences the weight-loss behaviors. In reviewing the literature, the melting point of KCl was 770 °C [35]; from this, we can infer that there was almost no weight loss of KCl in the range of 200–500 °C. Similarly, Jones et al. [36] indicated that KCl showed almost no weight loss in the range of 0–770 °C and a significant weight loss ranging from 770 to 950 °C with a weight loss of 70 wt.% at 950 °C. This is a crucial conclusion because if no interaction occurs between the two raw materials, they maintain their pyrolysis behaviors during the pyrolysis process. Fortunately, PSKCl-21, PSKCl-11, and PSKCl-12 had different pyrolysis behaviors (e.g., the position of the weight-loss peak) compared with PS (Figure 1b,d,f,h,j). Specifically, the significant weight-loss peak of PSKCl-21, PSKCl-11, and PSKCl-12 showed a shift to the left using the position of the maximum weight-loss peak of PS as the baseline. It follows that PS interacted with KCl during the pyrolysis process. In other words, the presence of KCl (the synergistic or catalytic) facilitates the decomposition of PS below 500 °C. Moreover, Jones et al. [36] suggested that the pyrolysis residue of KCl at 850 °C was ~77 wt.% at a heating rate of 20 °C/min. If this was used as the basis, assuming no interaction between PS and KCl, the pyrolysis residues of PSKCl-21, PSKCl-11, and PSKCl-1 were ~37.08, ~47.06, and ~57.04 wt.%, respectively (the pyrolysis residue of PS was obtained from Figure 1c). As shown in Figure 1c, the pyrolysis residues of PSKCl-21, PSKCl-11, and PSKCl-1 were 47.01, 55.77, and 68.95 wt.%, respectively. Notably, the pyrolysis residues of PSKCl-21, PSKCl-11, and PSKCl-12 obtained in Figure 1c were significantly higher than those of PSKCl-21, PSKCl-11, and PSKCl-12 calculated based on the above hypothesis. It follows that the presence of KCl is beneficial to increasing the yield of pyrolysis residue.

**Figure 2:**
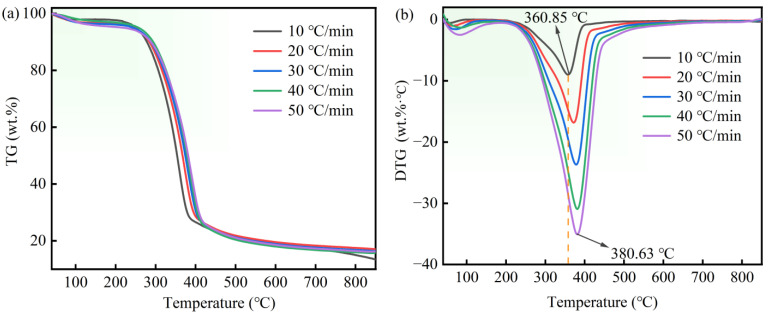
TG (**a**) and DTG (**b**) for PS at various heating rates.

The pyrolysis process at 10 °C/min was used as a case study for specific analysis. The loss of 1–4 wt.% of weight in the first stage was due to the removal of free water. The consecutive decomposition of the sample in the second stage was accompanied by a significant weight loss, leading to the formation of weight-loss peaks. For instance, ~69.36 wt.% weight loss was recorded for PS, ~41.46 wt.% weight loss was recorded for PSKCl-21, ~33.43 wt.% weight loss was recorded for PSKCl-11, and ~21.98 wt.% weight loss was recorded for PSKCl-12. In the third stage, the samples were deeply pyrolyzed, e.g., the pyrolysis residue of PS was diminished from 27.30 wt.% (the second stage) to 13.61 wt.% (the third stage). To further understand the effect of the heating rate on the pyrolysis behaviors, a systematic comparison of the TG/DTG curves at different heating rates (10, 20, 30, 40, and 50 °C/min) was carried out using PS as a case study (Figure 2).

The point maximum weight-loss rate of both TG and DTG curves shifted toward the high temperature. For instance, 360.85 °C recorded at 10 °C/min increased to 380.63 °C recorded at 50 °C/min (Figure 2b). This phenomenon could be attributed to the difference between the reference (viz., furnace) and sample temperatures, and is due to the heat and mass transfer limitations in the sample [37]. In addition, the biomasses’ own poor thermal conductivity equally leads to temperature gradients in the sample particles. The core temperature of biomass particles may be lower than the temperature of their surface [33,38]. Furthermore, the maximum weight loss in the DTG curve decreased as the heating rate increased, as influenced by mass transfer (with an increase in the heating rate, the heat transfer from the exterior to the interior of particles requires a longer time). As such, pyrolysis at higher heating rates was inadequate at the sample temperature, especially for biomass containing highly volatile components, a finding consistent with that of Lah and coworkers [39].

### 3.2. Theoretical and Experimental TG Curves

**Figure 3:**
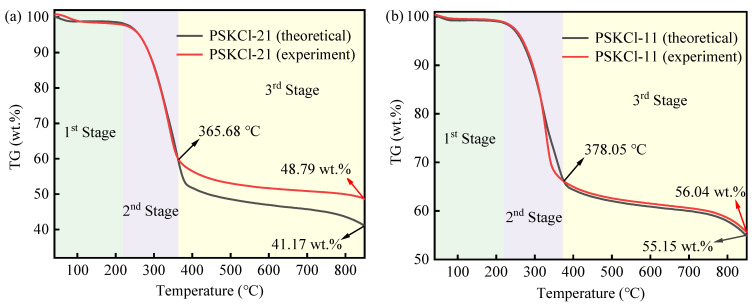
Theoretical and experimental TG curves for PSKCl-21 (**a**) and PSKCl-11 (**b**).

The two samples decomposed independently, implying that there is no interaction between the two samples during the pyrolysis procedure. Thus, the calculated values are the sum of individual sample values, which is proportional to their mixing weight ratios. Unfortunately, the thermogravimetric behaviors of KCl were not given as crucible fragmentation resulted under the action of high temperature. To further explain the interaction of KCl with PS, the theoretical TG curves of the samples need to be calculated. In this work, PSKCl-12 contained considerable amounts of KCl. Assuming no interaction between PS and KCl, it follows that the theoretical TG curves of PSKCl-11 and PSKCl-21 can be calculated from the TG curves of PS and PSKCl-12 with the following theoretical equations (PSKCl-11: TG_theoretical_ = 1/4 TG_PS_ + 3/4 TG_PSKCl-12_; PSKCl-21: TG_theoretical_ = 1/2 TG_PS_ + 1/2 TG_PSKCl-12_. Notably, TG_PS_ and TG_PSKCl-12_ stand for the experimental TG curves of PS and PSKCl-12, respectively). Overall, the theoretical TG curves resembled the experimental TG curves (Figure 3a,b). Notwithstanding this, the theoretical TG curves also exhibited pyrolysis behaviors that were quite different from the experimental TG curves. For instance, PSKCl-21 had 41.17 wt.% theoretical pyrolysis residue, which was significantly lower than its experimental pyrolysis residue (48.79 wt.%). A similar phenomenon was equally recorded in Figure 3b. Consequently, the presence of KCl decreased the resistance to biochar deposition. A plausible explanation for this result can be given as follows: at low temperatures, the solid KCl salt was uniformly mixed with the PS residue, whereas at elevated temperatures, the KCl salt melted and then the carbon block was sealed to prevent the intense combustion by the N_2_ atmosphere. By comparison, the theoretical and experimental TG curves recorded by PSKCl-21 and PSKCl-11 exhibited noticeable crossover points over 365.68 °C and 378.05 °C, respectively. In other words, the interaction between PS and KCl in PSKCl-11 and PSKCl-21 occurred after 365.68 °C and 378.05 °C, respectively. Notably, 378.05 °C > 365.68 °C; that is, the cross-point temperature gradually increased with improving the KCl content in the PS/KCl blends, which was related to the high melting point of KCl. It is worth noting that the equations used to estimate interactions in this work are not recommended if the thermogravimetric curves of individual samples can be determined. Despite the limitations of the equations used to assess the interactions in the present work, the equations suggest the existence of interactions between PS and KCl within a range.

### 3.3. Pyrolysis Mechanism

**Figure 4:**
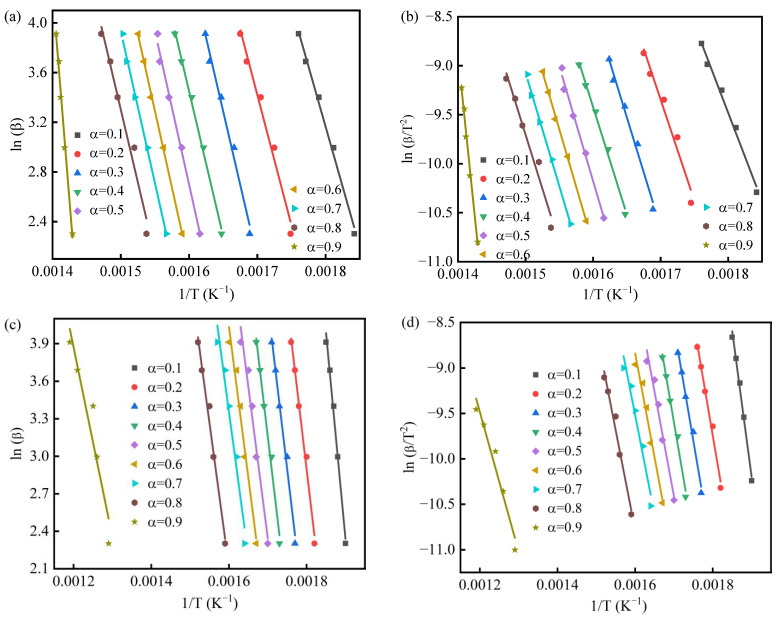

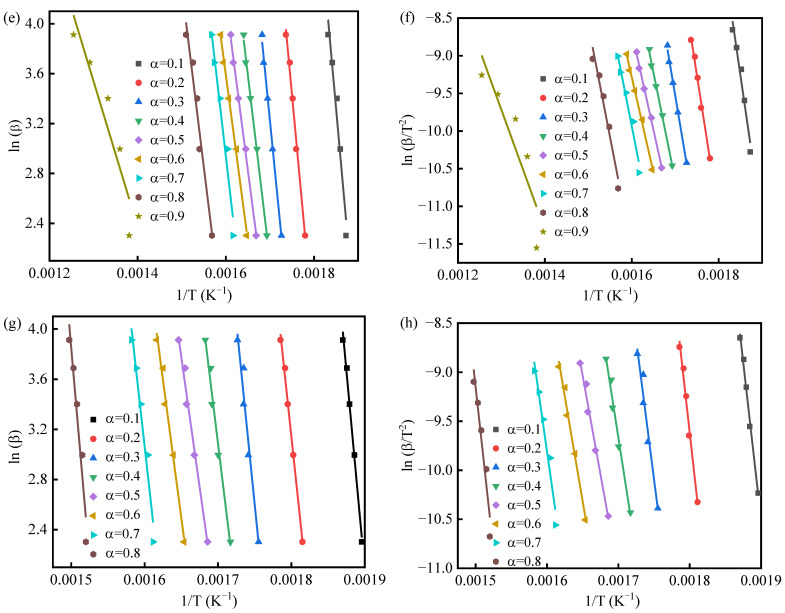
Linear plots for calculation of Arrhenius (E and A) for PS (**a**), PSKCl-21 (**c**), PSKCl-11 (**e**), and PSKCl-12 (**g**) applying the FWO method; linear plots for calculation of Arrhenius (E and A) for PS (**b**), PSKCl-21 (**d**), PSKCl-11 (**f**), and PSKCl-12 (**h**) employing the KAS method.

The TGA data can be analyzed to obtain the kinetic and thermodynamics (E, ΔG, ΔS, and ΔH) parameters in a transient process (i.e., the thermal decomposition of the solids). For instance, Mallick et al. [40] calculated the thermodynamics (E, ΔG, ΔS, and ΔH) parameters of biomass blends using TGA data. The results suggested that positive ΔG and ΔH were obtained for pyrolysis for all biomass blends. Similarly, Tabal et al. [29] obtained the thermodynamics (E, ΔG, ΔS, and ΔH) values of ficus wood and then indicated the thermodynamics parameters that were beneficial in assimilating the thermal decomposition of ficus wood for its use in bioenergy. Meanwhile, to gain physical insight into the interactions occurring in the co-pyrolysis of KCl and PS, TGA data of 10, 20, 30, 40, and 50 °C/ min were evaluated by two model-free (FWO and KAS) methods, respectively (Figure 4a–h). It is well known that samples have no significant weight loss after 550 °C, which produces considerable noise and leads to deviations in activation energy [41]. Consequently, the conversion α range for PS, PSKCl-11, and PSKCl-21 in this work was 0.1–0.9. Nevertheless, PSKCl-12 contained a large amount of KCl, which led to no significant weight loss after 500 °C; thus, the conversion α range of PSKCl-12 herein was 0.1–0.8. The plots of the FWO and KAS methods used to calculate the E values of PS, PSKCl-11, PSKCl-12, and PSKCl-21 showed similar trends and similar phenomena were recorded in the previous studies [42,43,44]. Table 1 lists the E value/conversion α relationship of various samples. Moreover, the mean E values recorded by PS, PSKCl-21, PSKCl-11, and PSKCl-12 were calculated using FWO and KAS methods: 208.24 kJ/mol and 223.31 kJ/mol for PS; 187.91 kJ/mol and 183.37 kJ/mol for PSKCl-21; 235.22 kJ/mol and 240.13 kJ/mol for PSKCl-11; and 421.96 kJ/mol and 436.78 kJ/mol for PSKCl-12. Thus, the E values recorded for PS were comparable to those of the reported samples characterized by similar compositions [45,46]. As an example, Yao and coworkers obtained the mean activation energies (α = 0.1–0.6) of 163, 167, and 162 kJ/mol recorded for bamboo, rice, and pinewood, respectively, by the FWO method [47]. Mallick and coworkers suggested the mean activation energies of 170.01 kJ/mol and 168.21 kJ/mol recorded for rice husk + sawdust blends evaluated by the KAS and FWO methods, respectively [40]. Notwithstanding this, it is vital to analyze the entire thermal degradation process since the E value relies on the conversion α.

**Figure 5:**
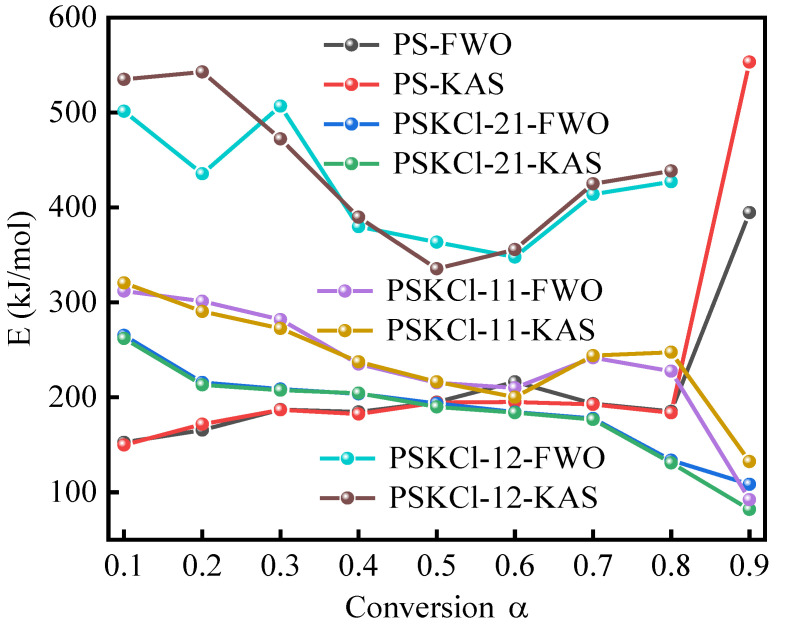
Activation energy/conversion α relationship of samples under various iso-conversional methods.

The E value trends of PS, PSKCl-21, PSKCl-11, and PSKCl-12 were determined using the FWO and KAS methods characterized by various conversion α (Figure 5). From Figure 5, it is observed that the values of E were different for all the conversion points. Variation in E values with degree of conversion (or pyrolysis) is essentially a consequence of the decomposition of different components of biomass with increasing temperature. Hemicellulose is known to degrade in the temperature range of 220–315 °C, while cellulose decomposes in the higher temperature range of 315–400 °C due to its crystalline nature. Lignin undergoes decomposition in the wide temperature range of 150–900 °C because of its complex structure. As discussed above, KCl showed basically no weight loss between room temperature and 770 °C. Accordingly, if there was no interaction between KCl and PS, the E value of the PS/KCl blends could be approximated as the E value of PS multiplied by the proportion of PS in the PS/KCl blends. As expected, the trend of E values with the conversion rate for PSKCl-12, PSKCl-11, and PSKCl-21 was not consistent with the trend of E values with the conversion rate for PS. From this, we can infer KCl interacts with PS in the pyrolysis temperature range. In general, the E profiles calculated for the FWO and KAS methods overlapped tightly, and a similar phenomenon was equally found in previous works [28,48]. The whole process can be divided into three stages. In the first stage (conversion α values: 0.1–0.3), the E values of PSKCl-11 and PSKCl-21 decreased with increasing conversion α, except for PSKCl-12 and PS. In the second stage (conversion α values: 0.3–0.7), the E values recorded for PS gradually increased as conversion α increased. Notably, at a conversion α of 0.7–0.8, the E values recorded for PSKCl-12 and PSKCl-11 were significantly higher than that of PS, whereas the E values of PSKCl-21 were lower than that of PS. As is known, the increase in E value indicates a more difficult degradation process, and this phenomenon can be ascribed to the generation of biochar characterized by a dense carbon-rich structure that possesses low degradation activity. From this, we can infer that when the KCl content in the PS/KCl blends was below 34%, the presence of KCl promoted the decomposition of PS. However, when the KCl content was above 50%, the presence of KCl increased the biochar yield. This phenomenon is crucial as it allows us to target-tune the yield of the three-phase product during biomass pyrolysis. The activation energy profiles of PS intersected with the activation energy profiles of PSKCl-21 when the conversion α was ~0.5. Similarly, the activation energy profiles of PS intersected with the activation energy profiles of PSKCl-11 when the conversion α was ~0.6; that is, as the KCl content in the PS/KCl blend increased, the intersection of its activation energy with the PS activation energy shifted to the right. The existence of KCl was more favorable for the degradation of PS in a range. The E values calculated by the FWO and KAS methods were very close to each other; so, in this work, the FWO method was used as a case study to investigate the master plots and pyrolysis thermodynamics.

**Figure 6:**
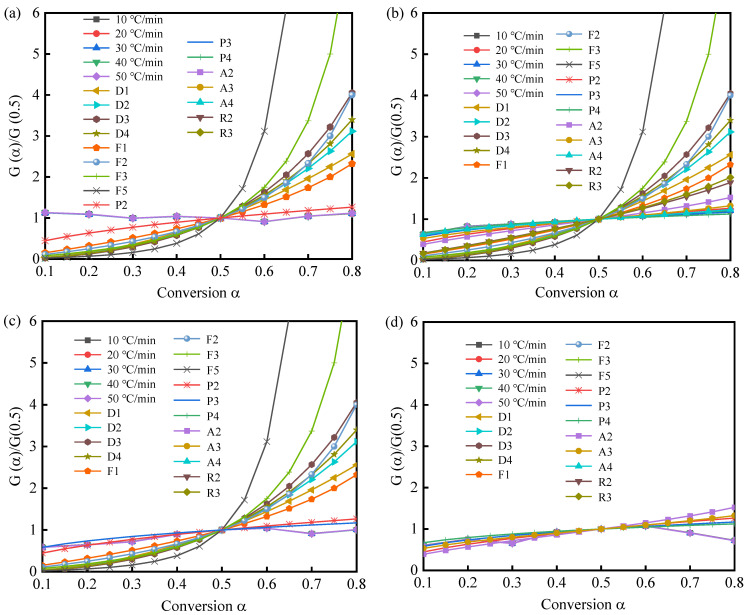
Theoretical and experimental master plots for the decomposition of (**a**) PS, (**b**) PSKCl-21, (**c**) PSKCl-11, and (**d**) PSKCl-12 at heating rates of 10, 20, 30, 40, and 50 °C/min.

The kinetic models are defined to understand the reaction kinetics. Furthermore, the integral master plots can be used to estimate the kinetic models when E is specified (Figure 6a–d). Additionally, Table 2 provides the kinetic models used for the reactions in non-homogeneous solid-state systems.

The similarity of the experimental master plots of $\frac{P\left(u\right)}{P\left(u_{0.5}\right)}$ vs. α for various heating rates (Figure 6a–d) suggests that the pyrolysis kinetics of PS, PSKCl-21, PSKCl-11, and PSKCl-12 can be described by a single kinetic model. The discussion was split into two parts in the present work, as exhibited below: (1) α ≤ 0.5, the experimental master plots of PSKCl-21, PSKCl-11, and PSKCl-12 decomposition more closely followed the P2, P3, and P3 profiles, respectively. Accordingly, the kinetic processes of PSKCl-21, PSKCl-11, and PSKCl-12 decomposition can be characterized by the nucleation model. Additionally, the experimental master plots recorded for PS decomposition more closely followed the D3 profile, which was similar to the experimental master plots for the reported biomasses (e.g., corn straw and grass) [49,50,51]. Notably, the D3 mechanism is related to the solid-state reaction, where diffusion acts as a key player [52]; (2) α ≥ 0.5, the experimental master plots for PS, PSKCl-21, PSKCl-11, and PSKCl-12 decomposition more closely followed the P2, P2, P3, and P3 profiles, respectively. Accordingly, the kinetic processes of PS, PSKCl-21, PSKCl-11, and PSKCl-12 decomposition can be characterized by the nucleation model.

To further understand the thermodynamics, A, ΔH, ΔG, and ΔS values were analyzed. The variation of A values with conversion α is listed in Table 3. Several factors, including complex composition and thermal behavior, are known to cause changes in A values [29]. The A values of the samples are as follows: 3.49 × 10^10^ to 9.54 × 10^30^ recorded for PS; 1.98 × 10^7^ to 2.90 × 10^21^ recorded for PSKCl-21; 5.18 × 10^5^ to 2.14 × 10^25^ recorded for PSKCl-11; and 6.68 × 10^30^ to 4.58 × 10^47^ recorded for PSKCl-12. Notably, PS possessed lower A values at a conversion α value of 0.1–0.8, indicating that PS had simpler composition than PSKCl-11, PSKCl-12, and PSKCl-21. These results agree with the fact that the composition of the PS/KCl blends was more complex compared to that of PS. Higher values of frequency factor (A ≥ 10^9^ s^−1^) essentially indicate a highly reactive system with a simple complex at different conversion rates. In addition, the A values were found to increase with conversion rates (α = 0.1–0.8), indicating increasing collision intensity between the reacting molecules with temperature [40]. Interestingly, a positive linear correlation between lnA values and E values is shown in Table 1 and Table 3. Similar findings were reported in previous literature [29].

**Figure 7:**
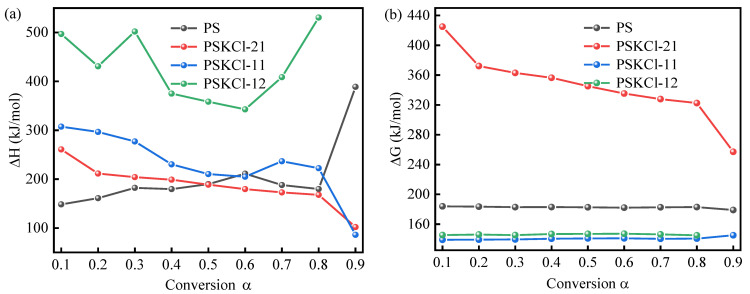

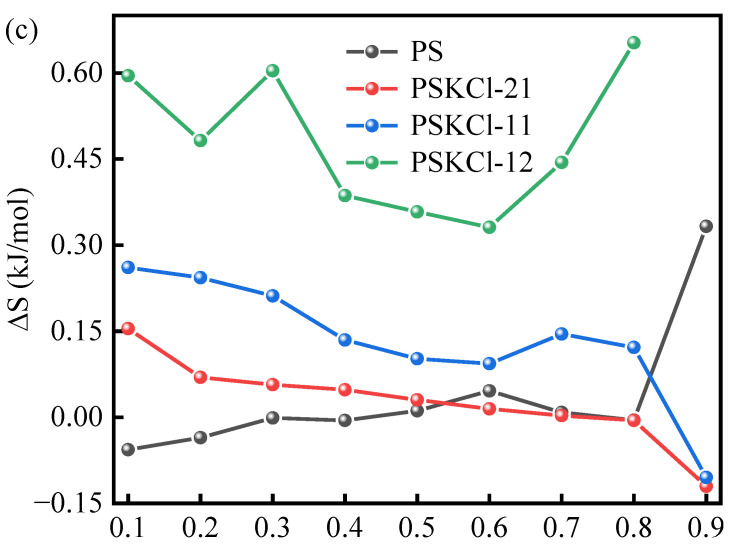
(**a**) ΔH, (**b**) ΔG, and (**c**) ΔS at various conversion α values.

Enthalpy (ΔH) is the total energy content of the system, and in the present context, it is essentially the internal energy of the PS/KCl blends. For the reaction system of biomass pyrolysis, the change in ΔH values represents the difference between the total energy of the formation of products and the reactants. That is, ΔH is the total energy consumed by the biomass for decomposition into various solid, liquid, and gaseous products. Similarly, ΔG and ΔS values represent the increase in total reaction energy during the formation of activated complexes and the degree of disorder associated with the production of activated complexes, respectively [53,54,55]. Figure 7a–c shows the ΔH, ΔG, and ΔS values at various conversion α values. PSKCl-12 and PSKCl-11 had higher ΔH values than PS at a conversion α value of 0.1–0.8 (Figure 7a). Meanwhile, the ΔH values of PSKCl-21 were higher than those of PS at α < 0.5. It is noteworthy that this trend corresponds to the change in activation energy. Despite this, the E and ΔH values showed several differences; for instance, the mean E and mean ΔH values of PS were 208.24 kJ/mol (the FWO method) and 203.18 kJ/mol, respectively. The smaller difference in energy shows that the products are formed thanks to the low potential energy, that is, the ease of activating complex formation (or the low potential barriers to activating complex formation) to convert the reactants into products. Except for PSKCl-21, the G values of the other samples showed insignificant changes (Table 4 and Figure 7b). As is known, the ΔG values indicate the quantity of energy that could be turned into usable energy through the pyrolysis of biomass. Remarkably, PS exhibited a mean ΔG value of 182.49 kJ/mol (Table 4). Based on this, it could be deduced that PS offers promising bioenergy potential via pyrolysis.

Several samples (i.e., PS, PSKCl-21, and PSKCl-11), except for PSKCl-12, had positive and negative ΔS values (Figure 7c). It is well known that a negative ΔS value demonstrates the change of the reaction system from a disordered state to an ordered state, which is crucial for converting biomass into biofuels and chemical feedstocks [56]. In addition, the lower ΔS values represent materials that recently underwent several physical and chemical variations, bringing them nearer to thermodynamic equilibrium. A positive value of ΔS indicates that the system is far from thermal equilibrium, which also affects the reactivity of the system. In conclusion, the comparative complexity of the thermal decomposition of PSKCl-21, PSKCl-11, and PSKCl-12 corresponds to the fact that they were compositionally complex.

### 3.4. Disorder Analysis

**Figure 8:**
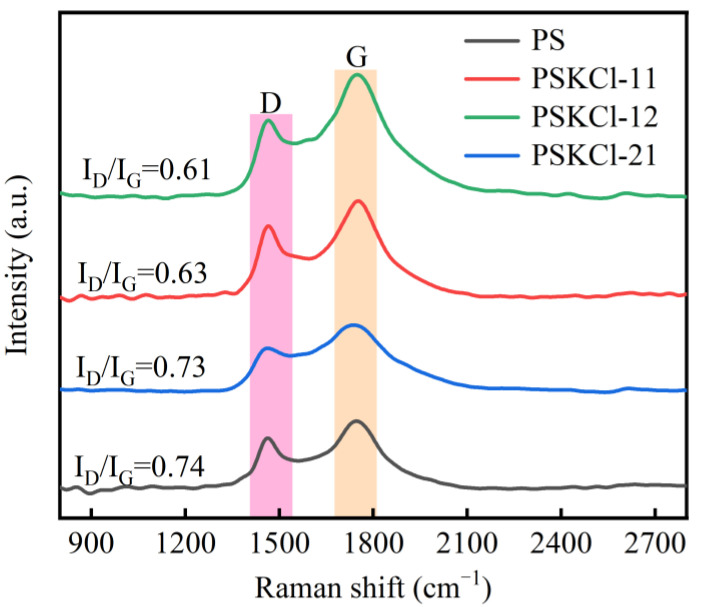
Raman spectra for samples.

Raman spectra (Figure 8) were applied to determine the defect or disorder information of pyrolysis residue. As is known, peaks at ~1340 cm^−1^ and ~1590 cm^−1^ represent the D-band (amorphous sp^3^ carbon) and G-band (ascribed to the graphitic sp^2^ carbon), respectively. Typically, the I_D_/I_G_ value is utilized to assess the disorder degree of samples [57]. The pre-oxidation inhibits the graphitization of carbon materials [58]. The graphite layers of the pre-oxidized samples tend to bend and twist randomly during subsequent carbonization rather than grow and rearrange, resulting in larger layer spacing [59]. Conversely, the I_D_/I_G_ values of PS, PSKCl-21, PSKCl-11, and PSKCl-12 were 0.74, 0.73, 0.63, and 0.61, respectively. Interestingly, Wang et al. [60] demonstrated that the carbon material structure, nitrogen species, and graphitization degree are effectively controlled by adjusting the KCl dosage and pyrolysis temperature. Notably, this work equally relates to the enhancement of graphitization on the pyrolysis of a zinc-containing ionic liquid. It follows that KCl does have a positive impact, but that does not mean that it would do the same on biomass (e.g., PS). In this context, it is desirable to investigate the effect of KCl on graphitization during biomass pyrolysis. In addition, Zhao et al. [61] indicated that manganese ions assisted in catalytic carbonization and precisely tuned the degree of graphitization. Encouragingly, the order of graphitization degrees for the samples was established: PSKCl-12 > PSKCl-11 > PSKCl-21 > PS. Notably, the I_D_/I_G_ values of PS and PSKCl-21 were higher than in the previous work (I_D_/I_G_: 0.71 [62]), implying that PS and PSKCl-21 possessed more disordered carbon. In addition, PSKCl-12 and PSKCl-11 possessed lower I_D_/I_G_ values than those of the previous work (I_D_/I_G_: 0.90 [62] and 0.97 [63]), suggesting that PSKCl-12 and PSKCl-11 had more ordered graphitic structures. More encouraging, with increasing KCl content in the PS/KCl blends, the graphitization of their pyrolysis residues gradually increased. When the pyrolysis temperature was over 700 °C (conversion α value > 0.8), the appropriate amount of KCl improved the graphitization of the pyrolysis residue by reducing the E, ΔS, and ΔH values of the PS/KCl blends. From this, we can infer that KCl can assist biochar graphitization. In conclusion, this work showed that the presence of KCl during biomass pyrolysis can improve the yield and graphitization of biochar. Of note is that this work did not examine the devolatilization of KCl, the preparation of porous carbon in a tube furnace, or the characterization of porous carbon, or discuss the effect of chlorine content on the electrical conductivity and adsorption properties of porous carbon; thus, the study does not indicate that the devolatilization of KCl, the preparation of porous carbon in a tube furnace, the characterization of porous carbon, or the discussion of the effect of chlorine content on the electrical conductivity and adsorption properties of porous carbon are not crucial. Notwithstanding its limitations, this study does provide a large amount of critical data for understanding the co-pyrolysis of PS with KCl.

## 4. Conclusions

In the present work, TGA was applied to probe the interaction mechanism of the PS/KCl blends. The PSKCl-21, PSKCl-11, and PSKCl-12 samples possessed various pyrolysis behaviors compared with PS. In addiiton, the cross-point temperature gradually increased with improving the KCl content in the PS/KCl blends, which was related to the high melting point of KCl. When the KCl content in the PS/KCl blends was below 34%, the presence of KCl promoted the decomposition of PS. However, when the KCl content was above 50%, the presence of KCl increased the biochar yield. Within a range, as the KCl content in the PS/KCl blend increased, the intersection of its activation energy with the PS activation energy shifted to the right. At lower conversion α values (α ≤ 0.5), the experimental master plots of PSKCl-21, PSKCl-11, and PSKCl-12 showed decomposition near the P2, P3, and P3 profiles, respectively. Notably, the lnA value was linearly positively related to the E value. Moreover, negative and positive ΔS values were calculated from the pyrolysis of PS, PSKCl-11, and PSKCl-21. In short, the present work indicated that the presence of KCl during biomass pyrolysis can enhance the yield and graphitization of biochar within a range.

## Figures and Tables

**Table 1:** Activation energy/conversion α relationship.

Conversion α	PS		PSKCl-21		PSKCl-11		PSKCl-12	
	FWO E (kJ/mol)	KAS E (kJ/mol)	FWO E (kJ/mol)	KAS E (kJ/mol)	FWO E (kJ/mol)	KAS E (kJ/mol)	FWO E (kJ/mol)	KAS E (kJ/mol)
0.1	152.52	149.81	265.27	262.03	311.86	320.55	501.38	534.96
0.2	165.55	171.68	215.58	213.06	301.23	290.39	435.48	542.65
0.3	187.10	186.79	208.74	207.48	281.86	272.59	506.78	472.18
0.4	184.58	182.44	203.65	204.36	235.12	237.38	379.85	389.82
0.5	194.97	194.61	193.57	189.97	215.34	216.39	363.42	335.48
0.6	216.37	195.00	184.51	183.87	210.02	200.25	347.79	355.70
0.7	193.28	192.50	177.82	176.66	241.73	243.86	413.86	424.96
0.8	185.18	183.76	133.73	130.97	227.65	247.45	427.12	438.52
0.9	394.61	553.18	108.32	81.94	92.16	132.35	n.d	n.d

Activation energy that could not be evaluated was marked with n.d.

**Table 2:** Kinetic models for reactions in heterogeneous solid-state systems.

Model		$\mathrm{Differential}\;\mathrm{Form}\;f\;(\alpha )=\frac{1}{k}\cdot \frac{d\alpha }{dt}$	Integral form G (α)
Nucleation models			
Power law	P2	2α^1/2^	α^1/2^
Power law	P3	3α^2/3^	α^1/3^
Power law	P4	4α^3/4^	α^1/4^
Avarami-Erofeyev	A2	2(1 − α)[−ln(1 − α)]^1/2^	[−ln(1 − α)]^1/2^
Avarami-Erofeyev	A3	3(1 − α)[−ln(1 − α)]^2/3^	[−ln(1 − α)]^1/3^
Avarami-Erofeyev	A4	4(1 − α)[−ln(1 − α)]^3/4^	[−ln(1 − α)]^1/4^
Geometrical contraction models			
Contracting area	R2	2(1 − α)^1/2^	[1 − (1 − α)^1/2^]
Contracting volume	R3	3(1 − α)^2/3^	[1 − (1 − α)^1/3^]
Diffusion models			
1-D diffusion	D1	1/2α	α^2^
2-D diffusion	D2	[−ln(1 − α)]^−1^	[(1 − α)ln(1 − α)] + α
3-D diffusion, Jander	D3	3(1 − α)^2/3^/[2(1 − (1 − α)^1/3^)]	[1 − (1 − α)^1/3^]^2^
Ginstling-Brounshtein	D4	3/[2((1 − α)^−1/3^ − 1)]	1 − (2α/3) − (1 − α)^2/3^
Reaction-order models			
First-order	F1	(1 − α)	−ln(1 − α)
Second-order	F2	(1 − α)^2^	(1 − α)^−1^ − 1
Third-order	F3	(1 − α)^3^	[(1 − α)^−2^ − 1]/2
Fifth-order	F5	(1 − α)^5^	[(1 − α)^−4^ − 1]/4

**Table 3:** Variation for pre-exponential factor with conversion α.

Conversion α	A (s^−1^)			
	PS	PSKCl-21	PSKCl-11	PSKCl-12
0.1	3.49 × 10^10^	2.90 × 10^21^	2.14 × 10^25^	3.65 × 10^44^
0.2	4.58 × 10^11^	1.11 × 10^17^	2.46 ×10^24^	4.74 × 10^38^
0.3	2.96 × 10^13^	2.49 × 10^16^	4.79 × 10^22^	1.11 × 10^45^
0.4	1.80 × 10^13^	8.69 × 10^15^	3.48 × 10^18^	4.99 × 10^33^
0.5	1.38 × 10^14^	1.07 × 10^15^	6.08 × 10^16^	1.68 × 10^32^
0.6	9.08 × 10^15^	1.64 × 10^14^	2.05 × 10^16^	6.68 × 10^30^
0.7	9.93 × 10^13^	4.10 × 10^13^	1.34 × 10^19^	5.52 × 10^36^
0.8	2.03 × 10^13^	1.54 × 10^13^	7.54 × 10^17^	4.58 × 10^47^
0.9	9.54 × 10^30^	1.98 × 10^7^	5.18 × 10^5^	n.d

A that could not be evaluated are marked with n.d.

**Table 4:** Variation in thermodynamic parameters (calculated by the FWO method) with conversion α.

Conversion α	PS			PSKCl-21			PSKCl-11			PSKCl-12		
	ΔH	ΔG	ΔS	ΔH	ΔG	ΔS	ΔH	ΔG	ΔS	ΔH	ΔG	ΔS
0.1	148.30	183.91	−5.65 × 10^−2^	260.87	424.86	15.44 × 10^−2^	307.42	139.07	26.11 × 10^−2^	496.99	145.39	59.51 × 10^−2^
0.2	161.12	183.48	−3.55 × 10^−2^	211.40	372.14	6.96 × 10^−2^	296.56	139.24	24.33 × 10^−2^	430.89	146.08	48.20 × 10^−2^
0.3	182.18	182.85	−0.11 × 10^−2^	204.03	362.90	5.69 × 10^−2^	277.05	139.57	21.15 × 10^−2^	502.03	145.33	60.37 × 10^−2^
0.4	179.53	182.92	−0.54 × 10^−2^	198.84	356.38	4.80 × 10^−2^	230.21	140.48	13.48 × 10^−2^	375.00	146.75	38.63 × 10^−2^
0.5	189.82	182.64	1.14 × 10^−2^	188.68	345.20	3.05 × 10^−2^	210.36	140.92	10.22 × 10^−2^	358.48	146.97	35.80 × 10^−2^
0.6	211.14	182.09	4.61 × 10^−2^	179.54	335.24	1.47 × 10^−2^	204.98	141.04	9.33 × 10^−2^	342.76	147.18	33.10 × 10^−2^
0.7	187.98	182.68	0.84 × 10^−2^	172.77	327.79	0.31 × 10^−2^	236.59	140.34	14.52 × 10^−2^	408.70	146.33	44.40 × 10^−2^
0.8	179.77	182.91	−0.50 × 10^−2^	167.81	322.41	−0.55 × 10^−2^	222.35	140.64	12.18 × 10^−2^	530.62	145.06	65.25 × 10^−2^
0.9	388.79	178.94	33.27 × 10^−2^	101.88	257.04	−12.02 × 10^−2^	86.14	145.16	−10.48 × 10^−2^	n.d	n.d	n.d

Herein, the units for ΔH, ΔG, and ΔS are all kJ/mol. Thermodynamic parameters that could not be evaluated are marked with n.d.

## Data Availability

Not applicable.

## Abbreviations

**Acronym**	**Meaning**
PS	Pine sawdust
PSKCl-21	The sample was obtained by mixing PS and KCl with a weight ratio of 2:1
PSKCl-11	The sample was obtained by mixing PS and KCl with a weight ratio of 1:1
PSKCl-12	The sample was obtained by mixing PS and KCl with a weight ratio of 1:2
TGA	Thermogravimetric analysis
A	Pre-exponential factor
ΔH	Enthalpy
ΔG	Gibbs free energy
ΔS	Entropy
FWO	Flynn-Wall-Ozawa
KAS	Kissinger-Akahira-Sunose
E	Activation energy
T	Absolute temperature
R^2^	Correlation coefficient
K	Boltzmann constant
T_m_	The peak temperature for the DTG curve
h	Plank constant
